# The dynamic interactome of human Aha1 upon Y223 phosphorylation

**DOI:** 10.1016/j.dib.2015.10.028

**Published:** 2015-11-06

**Authors:** Donald Wolfgeher, Diana M. Dunn, Mark R. Woodford, Dimitra Bourboulia, Gennady Bratslavsky, Mehdi Mollapour, Stephen J. Kron, Andrew W. Truman

**Affiliations:** a Department of Molecular Genetics and Cell Biology, The University of Chicago, Chicago, IL 60637, USA; b Department of Urology, SUNY Upstate Medical University, Syracuse, NY 13210, USA; c Department of Biochemistry and Molecular Biology, SUNY Upstate Medical University, Syracuse, NY 13210, USA; d Cancer Research Institute, SUNY Upstate Medical University, Syracuse, NY 13210, USA; e Department of Biological Sciences, University of North Carolina at Charlotte, Charlotte, NC 28223, USA

**Keywords:** Cancer, Hsp90, Interactome, Proteomics, Phosphorylation, Chaperones, Aha1, Co-chaperones

## Abstract

Heat Shock Protein 90 (Hsp90) is an essential chaperone that supports the function of a wide range of signaling molecules. Hsp90 binds to a suite of co-chaperone proteins that regulate Hsp90 function through alteration of intrinsic ATPase activity. Several studies have determined Aha1 to be an important co-chaperone whose binding to Hsp90 is modulated by phosphorylation, acetylation and SUMOylation of Hsp90 [1], [2]. In this study, we applied quantitative affinity-purification mass spectrometry (AP-MS) proteomics to understand how phosphorylation of hAha1 at Y223 altered global client/co-chaperone interaction [3]. Specifically, we characterized and compared the interactomes of Aha1–Y223F (phospho-mutant form) and Aha1–Y223E (phospho-mimic form). We identified 99 statistically significant interactors of hAha1, a high proportion of which (84%) demonstrated preferential binding to the phospho-mimic form of hAha1.

The mass spectrometry proteomics data have been deposited to the ProteomeXchange Consortium (http://proteomecentral.proteomexchange.org) via the PRIDE partner repository [4] with the dataset identifier PXD001737.

## Specifications table

**Table:** 

Subject area	*Biology*
More specific subject area	*Molecular chaperones, Mass spectrometry, phosphorylation*
Type of data	^*18*^*O Quant LC–MS/MS Mass spectrometry data*
How data was acquired	*Mass spectrometry.* Thermo Q-Exactive Orbitrap
Data format	*.Raw
Experimental factors	*HEK293 cells expressing either FLAG–hAha1–Y223E or FLAG–Aha1–Y223F*
Experimental features	*FLAG–hAha1–Y223E or FLAG–Aha1–Y223F complexes were purified by magnetic bead immunoprecipitation and processed by mass spectrometry*
Data source location	*The University of Chicago, Chicago, Illinois, USA*
Data accessibility	The mass spectrometry proteomics data have been deposited to the ProteomeXchange Consortium (http://proteomecentral.proteomexchange.org) via the PRIDE partner repository with the dataset identifier PXD001737.

**Value of the data**

• This data provides a comprehensive interactome of human Aha1.
• This study examines the Aha1 interactome quantitatively ±Y223 phosphorylation.
• Identifies important client and co-chaperone proteins that are specifically altered by Y223 phosphorylation.
• Demonstrates a novel method for regulating Hsp90 function, a key molecule in cancer proliferation.

## 1 Experimental design, materials and methods

HEK293 cell lines expressing either hAha1–Y223F–FLAG or Y223E–FLAG were grown in Dulbecco׳s modified Eagle׳s minimal essential medium (DMEM, Invitrogen) supplemented with 10% fetal bovine serum [1], [2], [3], [4]. All cell lines were propagated at 37 °C in an atmosphere containing 5% CO_2_. Protein extraction from both HEK293 cells was carried out using methods previously described [5]. For immunoprecipitation, mammalian cell lysates were incubated with anti-FLAG antibody conjugated magnetic beads (Sigma) for 2 h at 4 °C and washed 4 times with fresh lysis buffer (20 mM Tris (pH 7.5), 100 mM NaCl, 1 mM MgCl_2_, 0.1% NP40, Protease and Phosphatase inhibitor mini tablet, EDTA-free (Pierce). hAha1 complexes were eluted with FLAG peptide (Apex Bio).

## 2 LC–MS/MS data acquisition

### 2.1 Trypsin digestion of hAha1–FLAG complexes from SDS-PAGE gels

After hAha1 complexes were obtained, samples were processed as in [6], [7]. Purified hAha1–Y223F–FLAG or Y223E–FLAG complexes were loaded onto a 12% MOPS buffered SDS-PAGE gel (Invitrogen) and run for 10 min at 200 v resulting in a ~2 cm “gel plug”. The gel was stained with 25 mL Imperial Stain (Pierce) at room temperature, and destained overnight in dH_2_O at 4 °C. The gel plugs for each sample to be analyzed were excised by sterile razor blade, divided into 2 sections ~1 cm each, and chopped into ~1 mm^3^ pieces. Each section was washed in dH_2_O and destained using 100 mM NH_4_HCO_3_ pH 7.5 in 50% acetonitrile. A reduction step was performed by addition of 100 μl 50 mM NH_4_HCO_3_ pH 7.5 and 10 μl of 200 mM tris (2-carboxyethyl) phosphine HCl at 37 °C for 30 min. The proteins were alkylated by addition of 100 μl of 50 mM iodoacetamide prepared fresh in 50 mM NH_4_HCO_3_ pH 7.5 buffer, and allowed to react in the dark at 20 °C for 30 min. Gel sections were washed in water, then acetonitrile, and vacuum dried. Trypsin digestion was carried out overnight at 37 °C with 1:50–1:100 enzyme–protein ratio of sequencing grade-modified trypsin (Promega) in 50 mM NH_4_HCO_3_ pH 7.5, and 20 mM CaCl_2_. Peptides were extracted with 5% formic acid and vacuum dried.

### 2.2 Isotopic labeling of trypsin-digested hAha1–FLAG complexes

Peptide digests were reconstituted with 70 μl of Tris–HCl buffer solution (10 mM of Tris–HCl, 150 mM NaCl, 20 mM CaCl_2_, pH 7.6), vortexed for at least 20 min to reconstitute the peptide mixture, then split into two vials of 30 μl each (^16^O vial and ^18^O vial), and 10 μl was retained unlabeled and stored in −80 °C. In a separate vial Mag-Trypsin beads (Clontech) were prepared as follows. 30 μl Mag-trypsin beads per rxn (^16^O or ^18^O) were pooled and washed 3 times with 800 μl of Tris–HCl Buffer Solution, then brought back up in 30 μl/sample of Tris–HCl buffer, and aliquoted to a new 1.5 ml vial in the quantity of 30 μl, vortexing lightly after each aliquot to keep the Mag-trypsin beads in suspension. Using a magnetic rack, the Tris–HCl buffer was removed from the beads, and the 30 μl of sample digest from above was added to the beads and vacuum dried. 30 μl of either ^16^O H_2_O or 97% ^18^O H_2_O (Cambridge Isotopes Laboratories) was added to the respective ^16^O or ^18^O prepared Mag-Trypsin bead vial and vortexed for 20 min to reconstitute the peptide mixture, and allowed to exchange overnight at 37 °C. After ^18^O exchange, the solution was transferred to a new vial and any free trypsin in solution was inactivated with 1 mM PMSF for 30 min at 4 °C. For each sample section, the AHA-1 (E/F) digests were combined 1:1 as follows: Forward (FWD) Sample Set: (F)-^16^O:(E)-^18^O and Reversed (REV) Sample Set: (E)-^16^O:(F)-^18^O, dried and stored at −80 °C until analysis. Three biological replicate experiments were performed.

### 2.3 HPLC for mass spectrometry

All samples were re-suspended in Burdick & Jackson HPLC-grade water containing 0.2% formic acid (Fluka), 0.1% TFA (Pierce), and 0.002% Zwittergent 3–16 (Calbiochem), a sulfobetaine detergent that contributes the following distinct peaks at the end of chromatograms: MH^+^ at 392, and in-source dimer [2M+H^+^] at 783, and some minor impurities of Zwittergent 3–12 seen as MH^+^ at 336. The peptide samples were loaded to a 0.25 μl C_8_ OptiPak trapping cartridge custom-packed with Michrom Magic (Optimize Technologies) C8, washed, then switched in-line with a 20 cm by 75 μm C_18_ packed spray tip nanocolumn packed with Michrom Magic C18AQ, for a 2-step gradient. Mobile phase A was water/acetonitrile/formic acid (98/2/0.2) and mobile phase B was acetonitrile/isopropanol/water/formic acid (80/10/10/0.2). Using a flow rate of 350 nl/min, a 90 min, 2-step LC gradient was run from 5% B to 50% B in 60 min, followed by 50–95% B over the next 10 min, hold 10 min at 95% B, back to starting conditions and re-equilibrated.

### 2.4 LC–MS/MS analysis

The samples were analyzed via electrospray tandem mass spectrometry (LC–MS/MS) on a Thermo Q-Exactive Orbitrap mass spectrometer, using a 70,000 RP survey scan in profile mode, *m*/*z* 360–2000 Da, with lockmasses, followed by 10 MSMS HCD fragmentation scans at 17,500 resolution on doubly and triply charged precursors. Single charged ions were excluded, and ions selected for MS/MS were placed on an exclusion list for 60 s.

### 2.5 LC–MS/MS data analysis, statistical analysis

All LC–MS/MS *.raw Data files were analyzed with MaxQuant version 1.2.2, searching against the SPROT Human database using the following criteria: ^18^O heavy label was selected for quantitation with a min of 1 high confidence peptide to assign quantitation H/L ratio. Trypsin was selected as the protease with max miss cleavage set to 2. Carbamiodomethyl (C) was selected as a fixed modification. Variable modifications were set to Oxidization (M), Formylation (*n*-term), and Phosphorylation (STY). Orbitrap mass spectrometer was selected using an MS error of 20 ppm and a MS/MS error of 0.5 Da. 1% FDR cutoff was selected for peptide, protein, and site identifications.

Ratios were reported based on the MS level light and heavy peak areas determined by MaxQuant and reported in the proteinGroups.txt file as heavy/light or (hAha1–Y223E/hAha1–Y223F mutant). Proteins were removed from this results file if they were flagged by MaxQuant as “Contaminants”, “Reverse” or “Only identified by site”. Complete three biological replicates were performed, with each biological replicate split into two technical replicates (^18^O forward (FWD) labeling, and ^18^O reverse (REV) labeling). The abundance data from each biological replicate were normalized to the ratio of the bait protein in that run (e.g. normalized to the hAHA1–FLAG ratio). Light and Heavy peak intensities were analyzed in each run to determine protein hits that fell into the category of either hAha1–Y223E-only hits or hAha1–Y223F-only hits and retained if they confirmed to this state across all 6 runs. In the case of hAha1–Y223E-only or hAha1–Y223F-only protein hits, spectra counts can be used as a proxy for abundance as these would not of been assigned a quantitation ratio. This produced a list of hAha1 interactors and their respective quantitated changes between hAha1–Y223F and hAha1–Y223E.

Further statistical analysis was performed using the R statistical package (http://www.r-project.org/). Proteins with three out of the six observations were retained. Missing values were imputed using row mean imputation. An ANOVA test was then performed to identify proteins that indicate significant variability (*P*-value<0.05) between biological replicates within each group. The mass spectrometry proteomic data have been deposited to the ProteomeXchange Consortium (http://proteomecentral.proteomexchange.org) via the PRIDE partner repository (REF A) with the dataset identifier PXD001737.
