# Supplementary material for: The dynamic interactome of human Aha1 upon Y223 phosphorylation
Source: Data Brief. 2015 Nov 6;5:752–5. doi: 10.1016/j.dib.2015.10.028 (PMC4659802; doi:10.1016/j.dib.2015.10.028)
Supplement: Supplementary file 2 — Supplementary material [file mmc2.pdf]

Conflict of Interest Form

None
